# Supplementary material for: Comparative genomics provides new insights into the diversity, physiology, and sexuality of the only industrially exploited tremellomycete: Phaffia rhodozyma
Source: BMC Genomics. 2016 Nov 9;17:901. doi: 10.1186/s12864-016-3244-7 (PMC5103461; doi:10.1186/s12864-016-3244-7)
Supplement: Additional file 6: — List of orphan genes with links to PFAM (related to Additional file 1: Table S1). (ZIP 1428 kb) [file 12864_2016_3244_MOESM6_ESM.zip › BLAST_HTML_FTR/G00488_P.html]

BLAST Search Results


```
BLASTP 2.2.27+


Reference:
Stephen F. Altschul, Thomas L. Madden, Alejandro A. Schäffer,
Jinghui Zhang, Zheng Zhang, Webb Miller, and David J. Lipman (1997),
"Gapped BLAST and PSI-BLAST: a new generation of protein database
search programs", Nucleic Acids Res. 25:3389-3402.


Reference for
composition-based statistics:
Alejandro A. Schäffer, L. Aravind, Thomas L. Madden, Sergei
Shavirin, John L. Spouge, Yuri I. Wolf, Eugene V. Koonin, and
Stephen F. Altschul (2001), "Improving the accuracy of PSI-BLAST
protein database searches with composition-based statistics and
other refinements", Nucleic Acids Res. 29:2994-3005.


Database: nr
           71,551,133 sequences; 26,053,659,533 total letters


Query= G00488_P

Length=152
                                                                      Score     E
Sequences producing significant alignments:                          (Bits)  Value

emb|CED82788.1|  hypothetical protein [Xanthophyllomyces dendrorh...   304    3e-103
emb|CBI36930.3|  unnamed protein product [Vitis vinifera]             37.4    3.7   
ref|XP_002270132.1|  PREDICTED: uncharacterized protein LOC100267...  37.4    4.7   
ref|XP_003836077.1|  hypothetical protein LEMA_P054180.1 [Leptosp...  37.0    5.2   
emb|CAN73724.1|  hypothetical protein VITISV_016652 [Vitis vinifera]  37.0    6.3   


 >emb|CED82788.1| hypothetical protein [Xanthophyllomyces dendrorhous]
Length=151

 Score =  304 bits (779),  Expect = 3e-103, Method: Compositional matrix adjust.
 Identities = 151/151 (100%), Positives = 151/151 (100%), Gaps = 0/151 (0%)

Query  1    MPRWNNWYRPSDEPPFIKFLLGISVAISTIFILLTSFVLCCWIVDLVVYEVQAAGKLCLW  60
            MPRWNNWYRPSDEPPFIKFLLGISVAISTIFILLTSFVLCCWIVDLVVYEVQAAGKLCLW
Sbjct  1    MPRWNNWYRPSDEPPFIKFLLGISVAISTIFILLTSFVLCCWIVDLVVYEVQAAGKLCLW  60

Query  61   DESGGVTCFWRFFVGTYKHIKLTLGGTTGISPSLLEDSPASVSAAAAAVVSRSGTSAGDG  120
            DESGGVTCFWRFFVGTYKHIKLTLGGTTGISPSLLEDSPASVSAAAAAVVSRSGTSAGDG
Sbjct  61   DESGGVTCFWRFFVGTYKHIKLTLGGTTGISPSLLEDSPASVSAAAAAVVSRSGTSAGDG  120

Query  121  YEWRANESWPVGLSLGGLGASAGAARGRQAS  151
            YEWRANESWPVGLSLGGLGASAGAARGRQAS
Sbjct  121  YEWRANESWPVGLSLGGLGASAGAARGRQAS  151


>emb|CBI36930.3| unnamed protein product [Vitis vinifera]
Length=620

 Score = 37.4 bits (85),  Expect = 3.7, Method: Composition-based stats.
 Identities = 21/61 (34%), Positives = 31/61 (51%), Gaps = 3/61 (5%)

Query  82   LTLGGTTGISPSLLED---SPASVSAAAAAVVSRSGTSAGDGYEWRANESWPVGLSLGGL  138
            L L G  GISPS+  D   +P  VSA   + V++  TS+G  +++     WP G     +
Sbjct  4    LCLPGIHGISPSISVDVRANPTQVSAVGRSTVAQKTTSSGFSFKYSLKSLWPGGKGYYAI  63

Query  139  G  139
            G
Sbjct  64   G  64


>ref|XP_002270132.1| PREDICTED: uncharacterized protein LOC100267577 [Vitis vinifera]
Length=717

 Score = 37.4 bits (85),  Expect = 4.7, Method: Composition-based stats.
 Identities = 21/61 (34%), Positives = 31/61 (51%), Gaps = 3/61 (5%)

Query  82   LTLGGTTGISPSLLED---SPASVSAAAAAVVSRSGTSAGDGYEWRANESWPVGLSLGGL  138
            L L G  GISPS+  D   +P  VSA   + V++  TS+G  +++     WP G     +
Sbjct  4    LCLPGIHGISPSISVDVRANPTQVSAVGRSTVAQKTTSSGFSFKYSLKSLWPGGKGYYAI  63

Query  139  G  139
            G
Sbjct  64   G  64


>ref|XP_003836077.1| hypothetical protein LEMA_P054180.1 [Leptosphaeria maculans JN3]
 emb|CBX92712.1| hypothetical protein LEMA_P054180.1 [Leptosphaeria maculans JN3]
Length=828

 Score = 37.0 bits (84),  Expect = 5.2, Method: Composition-based stats.
 Identities = 23/51 (45%), Positives = 28/51 (55%), Gaps = 1/51 (2%)

Query  83   TLGGTTGISPSLLEDSPASVSAAAAAVVSRSGTSAGDGYEWRANESW-PVG  132
            TLG  TGI P  ++   A +S A +   SRSG S  + Y  R  ESW PVG
Sbjct  608  TLGTPTGILPHQVQPMQAELSRANSNCSSRSGVSGHNDYYQRPVESWTPVG  658


>emb|CAN73724.1| hypothetical protein VITISV_016652 [Vitis vinifera]
Length=740

 Score = 37.0 bits (84),  Expect = 6.3, Method: Composition-based stats.
 Identities = 21/61 (34%), Positives = 31/61 (51%), Gaps = 3/61 (5%)

Query  82   LTLGGTTGISPSLLED---SPASVSAAAAAVVSRSGTSAGDGYEWRANESWPVGLSLGGL  138
            L L G  GISPS+  D   +P  VSA   + V++  TS+G  +++     WP G     +
Sbjct  4    LCLPGIHGISPSISVDVRANPTQVSAVGRSTVAQKTTSSGFSFKYSLKSLWPGGKGYYAI  63

Query  139  G  139
            G
Sbjct  64   G  64


Lambda      K        H        a         alpha
   0.324    0.138    0.460    0.792     4.96 

Gapped
Lambda      K        H        a         alpha    sigma
   0.267   0.0410    0.140     1.90     42.6     43.6 

Effective search space used: 639134211780


  Database: nr
    Posted date:  Sep 23, 2015 12:05 AM
  Number of letters in database: 26,053,659,533
  Number of sequences in database:  71,551,133


Matrix: BLOSUM62
Gap Penalties: Existence: 11, Extension: 1
Neighboring words threshold: 11
Window for multiple hits: 40
```
